# Knowledge, Attitude, and Practices of Healthcare Workers Regarding the Use of Face Mask to Limit the Spread of the New Coronavirus Disease (COVID-19)

**DOI:** 10.7759/cureus.7737

**Published:** 2020-04-20

**Authors:** Jagdesh Kumar, Muhammad Soughat Katto, Adeel A Siddiqui, Badaruddin Sahito, Muhammad Jamil, Nusrat Rasheed, Maratib Ali

**Affiliations:** 1 Orthopaedic Surgery, Dow University of Health Sciences, Karachi, PAK

**Keywords:** healthcare workers, face mask, coronavirus, covid-19

## Abstract

Introduction

Many countries including Pakistan are currently using face masks in their pandemic control plans. Being highly prevalent, the correct use of these masks is particularly important, as an incorrect use and disposal may actually increase the rate of transmission. The purpose of this study was to investigate the knowledge, attitude, and practices of healthcare workers (HCWs) in wearing a surgical face mask to limit the spread of the new coronavirus disease 2019 (COVID-19).

Materials and Methods

This survey was conducted by interviewing HCWs using a questionnaire consisting of the basic demographic characteristics, and the knowledge, attitude, and practices regarding the use of surgical face mask to limit the new COVID-19 exposure. Each correct answer was scored 1 and each incorrect answer scored 0. The total number of questions was 16, and the final score was calculated and then labeled according to the percentage (out of 16) of correct responses as good (>80%), moderate (60-80%), and poor (<60%).

Results

A total of 392 participants with a mean age of 42.37 ± 13.34 years (341 males and 51 females) were included in the study. The overall final results were good in 138 (35.2%), moderate in 178 (45.4%), and poor in 76 (19.3%). Around 43.6% of participants knew about the correct method of wearing the masks, 68.9% knew that there are three layers, 53% stated that the middle layer act as a filter media barrier, and 75.5% knew the recommended maximum duration of wearing it. The majority (88.2%) of participants knew that a cloth face mask is not much effective, around 79.8% knew that used face mask cannot be re-used, and 44.8% knew about the yellow-coded bag for disposal.

Conclusions

Knowledge, attitude, and practice of HCWs regarding the use of face masks were found to be inadequate. Studied HCWs had a positive attitude but moderate-to-poor level of knowledge and practice regarding the use of face mask. HCWs and general public awareness campaigns regarding the proper use of face mask by utilizing all social media available resources would be helpful during this pandemic.

## Introduction

Coronavirus disease 2019 (COVID-19) is a respiratory illness caused by severe acute respiratory syndrome coronavirus 2 (SARS-CoV-2), which first emerged in China in December 2019, and has since spread to most countries around the world, resulting in the 2019-2020 coronavirus pandemic [1-3]. The virus primarily spreads between people through respiratory droplets, which are produced when an infected person coughs or sneezes, or by touching contaminated surfaces or objects and then touching their own mouth, nose, or possibly their eyes. The risk of getting severe COVID-19 is higher in health care workers (HCWs) who are in close contact with confirmed COVID-19 cases. The latest figures show thousands of HCWs getting infected with a large percentage of them dying [4].

In order to minimize risk, HCWs are required to follow accepted infection control practices. Aside from hand hygiene, one of the infection control measures is the routine use of a face mask. Many countries including Pakistan are currently using face masks in their pandemic plans. Face mask works by providing a physical barrier between the mouth and nose of the wearer and potential contaminants in the immediate environment [5].

In resource-limited settings, where the incidence of infectious disease is high and the environmental conditions of hospitals are often poor, hospitals may rely heavily on a face mask to protect medical staff against COVID-19 and to prevent cross-contamination among patients and HCWs. The use of a face mask among HCWs is strongly recommended by the World Health Organization (WHO) and the Centers for Disease Control and Prevention (CDC) as a standard for transmission-based precaution [6,7]. Moreover, the correct use of these masks is particularly important especially during this time when its use is becoming highly prevalent [8].

The WHO states that incorrect use and disposal of this mask may actually increase the rate of transmission. If you wear a mask, then you must know how to use it and discard it properly [7]. There is evidence that the HCWs have inadequate knowledge and poor practice regarding the use of surgical mask [9]. The purpose of this study was to investigate the knowledge, attitude, and practices of HCWs in wearing a face mask particularly a standard surgical face mask to limit the spread of COVID-19.

## Materials and methods

This cross-sectional community-based survey was conducted at the Department of Orthopedic Surgery, Dr. Ruth K. M. Pfau Civil Hospital, affiliated to Dow University of Health Sciences, Karachi, Pakistan, in March 2020 (one month).

The study participants were HCWs, that is, consultant, medical officer, postgraduate trainee, house officer, and paramedical staff. A convenient sampling method was used and a sample size of 384 was calculated, considering 5% precision, 95% confidence interval, and 52% as the correct practice of using face masks [5]. Keeping a minimum sample size of 384 in mind, a total of 392 patients were registered in the study duration.

The study was conducted by interview using a semi-structured questionnaire. The questionnaire was developed with the help of previous literature on the proper use of surgical face mask and the guidelines of the Centre for Health Protection and the CDC and consisted of two parts:(1) basic demographic characteristics (age, gender, job designation), and (2) knowledge, attitude, and practices regarding the use of a face mask to limit COVID-19 exposure [5,10,11]. Prior to the inception of the study, the nature and purpose of the study were explained to each respondent, and informed consent was obtained. For the convenience of analyses, each correct response in the knowledge category, good practice, or positive attitude was scored 1, and each incorrect response, bad practice, or negative attitude was scored 0. The total number of questions was 16, and the final score was calculated and then labeled according to the percentage (out of 16) of correct responses as good (>80%), moderate (60-80%), and poor (<60%).

The information obtained from the participants was entered and analyzed using Statistical Package for the Social Sciences (SDSS) Statistics for Windows, Version 22.0 (IBM Corp., Armonk, NY, USA). Mean with standard deviations were calculated for age and frequency with percentages for categorical variables.

## Results

A total of 392 participants were registered with a mean age of 42.37 ± 13.34 years, out of which 341 (86.9%) were males and 51 (13%) were females. Around 66 (16.8%) were consultants, 91 (23.2%) were medical officers, 117 (29.8%) were postgraduate trainees, 30 (7.7%) were house officers, and 88 (22.4%) were paramedical staffs.

When the knowledge was assessed, 56.4% knew the correct way of wearing a surgical mask, 68.9% knew that there are three layers in a surgical mask, and 53% knew how to identify the correct filter media barrier. Around 64.8% of participants knew the correct efficiency of masks that can actually protect against COVID-19, whereas 75.6% were aware of the maximum duration of wearing a face mask. When the study participants were asked about the extent to which surgical mask should cover, 74.7% answered correctly and 92% correctly reported the purpose of the metal strip (Table 1).

**Table 1:** Knowledge about the correct usage of masks BFE, bacterial filtration efficiency; PFE, particle filtration efficiency

Statement	Response	n	%
Which is the correct way of using surgical face mask to protect against COVID-19	White side facing out	171	43.6
	White side facing in (correct)	221	56.4
How many layers are there in a surgical mask	Two	97	24.7
	Three (correct)	271	68.9
	Four	24	6.1
Can wearing a surgical mask protect you from COVID-19	Yes	278	70.9
	No	114	29
Which layer acts as a filter media barrier	First layer	130	33.1
	Middle layer (correct)	208	53
	Last layer	53	13.7
Which type of masks actually protect against COVID-19	95% BFE and PFE (correct)	254	64.8
	97% BFE and PFE	06	1.5
	91% BFE and PFE	15	3.8
	99% BFE and PFE	117	29.8
How long can you wear a surgical mask	8 hours (correct)	296	75.6
	4 hours	56	14.3
	2 hours	40	10.2
	1 hour	0	0
For proper wearing, to which extent the surgical mask should cover?	Nose only	17	4.3
	Nose and mouth	82	20.9
	Nose, mouth, and chin (correct)	293	74.7
What is the purpose of the metal strip on a surgical mask	No purpose	18	4.6
	To fit on the nose (correct)	361	92
	To fit on the chin	13	3.3
Is the cloth facial mask as effective as a regular surgical facial mask	Yes	46	11.7
	No (correct)	346	88.2

As shown in Table 2, on evaluating the correct practices, 13.8% of participants used to remove the mask while talking to the patient, 20.2% reused the mask, and 44.9% correctly used the yellow-coded bag for disposal of the face mask. Around 93.9% of participants had a practice of wearing masks in clinics and 94.6% had a practice of wearing masks in hospital premises. Around 88.5% of participants were confident enough about their knowledge and practices of correctly wearing the face masks.

**Table 2:** Practices and attitude about the correct usage of masks

Statement	Response	n	%
Practices			
During clinics, if there is a need to talk to the patient, will you remove your mask	Yes	54	13.8
	No	338	86.2
If you are not sick, do you store the used surgical mask in a bag for later use	Yes	79	20.2
	No	313	79.8
Do you wear a mask in public places to protect yourself against COVID-19	Yes	368	93.9
	No	24	6.1
Do you wear a mask in hospital premises to protect yourself against COVID-19	Yes	371	94.6
	No	21	5.3
In which color-coded bag you dispose of your mask	Red-coded bag	116	29.6
	Yellow-coded bag	176	44.9
	Blue-coded bag	39	9.9
	Black-coded bag	61	15.6
Attitude			
Are you confident enough to know the correct steps of wearing a face mask	Yes	347	88.5
	No	45	11.47

The overall final results were good in 138 (35.2%), moderate in 178 (45.4%), and poor in 76 (19.3%).

## Discussion

Face masks are used as a protective barrier to reduce the risk of transmission of microorganisms between patients, HCWs, and the environment [12]. However, in order for face masks to provide effective protection, the HCWs must have an intimate knowledge of wearing and disposing of those. In this study, 88.5% of participants thought that they knew the proper steps of wearing a surgical face mask; however, only 35% obtained a good score by answering the procedural questions correctly. These results may be because of its simplest design, which leads many participants to mistakenly assume that they know the proper steps of wearing it.

There was higher male participation in our study (86.9%) compared with female participation (13%). This finding can be attributed to the higher male enrolment in our institution.

In this study, 64.7% of participants obtained an overall moderate-to-poor score regarding the correct usage of a surgical face mask. This low knowledge and practice may be because of recently circulating messages on social media claiming the proper way to wear the three-layered surgical mask, like “colored side facing out if you are sick, and the white side facing out if you want to ‘stop the germs from getting in’”.

This is, however, false and misleading, according to Nawhen, a columnist for Medical Mythbusters Malaysia, a non-governmental organization that works to counter myths and inaccurate facts on medical matters; the correct way to wear a surgical mask is by wearing the colored side facing out independent of your health status. The outer colored layer is hydrophobic or is a fluid-repelling layer and its main function is to prevent germs from sticking to it, whereas the inner one is a hydrophilic layer that absorbs moisture from the air we breathe out. If you wear it the other way round, the moisture from the air will stick onto it, thus making it easier for germs to stay there. There is a middle layer that actually filters the microorganism [13].

Cloth mask, re-use of a surgical mask, and its extended use are commonly seen in Pakistan during the extended outbreak of the COVID-19 pandemic. It is highly unlikely for low-income countries that they will be able to provide disposable face masks for that extended period of time and may have to ration the use of these products. In this study, around 88.2% HCWs agreed that cloth mask is not as effective as a regular surgical mask and about 79.8% knew that used surgical face mask cannot be re-used. Around 75.6% knew the correct maximum duration of using it. Other studies also highlighted similar findings concluding that cloth mask, re-use, and extended use of mask makes it ineffective, still HCWs are sometimes forced to do it due to the increasing shortage of these masks. We observed that wearing the same mask without removing it between patient encounters and disposing it properly at the end of the day is better than re-using it. Still if re-using it due to shortage, it is better to fold the mask in such a way that the outer contaminated surface is held inward followed by storing it in a clean sealable paper bag or container [14-16].

There is not enough evidence to prove that wearing a surgical mask protects every person from COVID-19. The WHO currently recommended that only HCWs and people who are ill and those who are caring for the ill need to wear a mask to protect themselves from COVID-19. However, in low-income countries like Pakistan, where the incidence of infectious disease is high and the hospital environmental conditions are often poor, our HCWs rely almost entirely on a face mask to limit the spread of COVID-19 [17].

The WHO established a color-coded bin system for proper disposal of biomedical waste in hospitals [18]. However, when it was asked from our participants, 44.9% disposed it in the yellow-coded bag for disposal of face mask; this shows poor knowledge of HCWs regarding the safe disposal of biomedical waste.

Some of the limitations of this study include the cross-sectional nature of study design limited to a single governmental hospital. Further longitudinal studies should be carried out on a larger sample size, and both private and government hospitals should be included before the results could be generalized. Moreover, different types of masks can be compared.

## Conclusions

Knowledge, attitude, and practice of HCWs regarding the use of surgical face masks were found to be inadequate. Studied HCWs had a positive attitude but moderate-to-poor level of knowledge and practice regarding the use of surgical face mask. HCWs and general public awareness campaigns regarding the proper use of face mask by utilizing all social media available resources would be helpful during this pandemic.
